# Thyroid Autoimmunity and SARS-CoV-2 Infection

**DOI:** 10.3390/jcm12196365

**Published:** 2023-10-05

**Authors:** Poupak Fallahi, Giusy Elia, Francesca Ragusa, Sabrina Rosaria Paparo, Armando Patrizio, Eugenia Balestri, Valeria Mazzi, Salvatore Benvenga, Gilda Varricchi, Laura Gragnani, Chiara Botrini, Enke Baldini, Marco Centanni, Clodoveo Ferri, Alessandro Antonelli, Silvia Martina Ferrari

**Affiliations:** 1 Department of Translational Research and New Technologies in Medicine and Surgery, University of Pisa, 56126 Pisa, Italy; poupak.fallahi@unipi.it (P.F.); sabrinapaparo@gmail.com (S.R.P.); laura.gragnani@unipi.it (L.G.); 2 Department of Surgery, Medical and Molecular Pathology and of Critical Area, University of Pisa, 56126 Pisa, Italy; giusy.elia@phd.unipi.it (G.E.); francesca.ragusa@phd.unipi.it (F.R.); balestri90@gmail.com (E.B.); mazzivaleria@gmail.com (V.M.); chiara.botrini@gmail.com (C.B.); 3 Department of Emergency Medicine, Azienda Ospedaliero-Universitaria Pisana, 56126 Pisa, Italy; armando.patrizio@ao-pisa.toscana.it; 4 Department of Clinical and Experimental Medicine—Endocrinology, University of Messina, 98122 Messina, Italy; s.benvenga@live.it; 5 Master Program on Childhood, Adolescent and Women’s Endocrine Health, University of Messina, 98122 Messina, Italy; 6 Interdepartmental Program of Molecular & Clinical Endocrinology and Women’s Endocrine Health, University Hospital Policlinico “G. Martino”, 98124 Messina, Italy; 7 Department of Translational Medical Sciences, University of Naples Federico II, 80131 Naples, Italy; gildanet@gmail.com; 8 Center for Basic and Clinical Immunology Research, University of Naples Federico II, 80131 Naples, Italy; 9 World Allergy Organization Center of Excellence, University of Naples Federico II, 80131 Naples, Italy; 10 Institute of Experimental Endocrinology and Oncology “Gaetano Salvatore”, National Research Council, 80131 Naples, Italy; 11 Department of Experimental Medicine, “Sapienza” University of Rome, 00185 Rome, Italy; enke.baldini@uniroma1.it; 12 Department of Medico-Surgical Sciences and Biotechnologies, Endocrinology Section, ‘‘Sapienza’’ University of Rome, 00185 Rome, Italy; marco.centanni@uniroma1.it; 13 Endocrine Unit, Azienda Unità Sanitaria Locale (AUSL) Latina, 04100 Latina, Italy; 14 Rheumatology Unit, School of Medicine, University of Modena and Reggio Emilia, 41100 Modena, Italy; clferri@unimore.it; 15 Rheumatology Clinic ‘Madonna Dello Scoglio’ Cotronei, 88836 Crotone, Italy; 16 Department of Clinical and Experimental Medicine, University of Pisa, 56126 Pisa, Italy; silvia.ferrari@unipi.it

**Keywords:** COVID-19, SARS-CoV-2 infection, autoimmune thyroid diseases, subacute thyroiditis, thyrotoxicosis

## Abstract

The severe acute respiratory syndrome coronavirus 2 (SARS-CoV-2), the etiological culprit of COronaVIrus Disease 19 (COVID-19), can enter the cells via the angiotensin-converting enzyme 2 (ACE2) receptor, which has been found in several tissues including in endocrine organs, such as the ovaries, testes, pancreas, and thyroid. Several thyroid disorders have been associated with SARS-CoV-2 infection [subacute thyroiditis (SAT), thyrotoxicosis, and non-thyroidal illness syndrome (NTIS)] and, in part, they are believed to be secondary to the local virus replication within the gland cells. However, as documented for other viruses, SARS-CoV-2 seems to interfere with several aspects of the immune system, inducing the synthesis of autoantibodies and triggering latent or new onset autoimmune disease (AID), including autoimmune thyroid disease (AITD), such as Hashimoto Thyroiditis (HT) and Graves’ disease (GD). Several mechanisms have been hypothesized to explain this induction of autoimmunity by SARS-CoV-2 infection: the immune system hyper-stimulation, the molecular mimicry between the self-antigens of the host and the virus, neutrophils extracellular traps, and finally, the virus induced transcriptional changes in the immune genes; nonetheless, more evidence is needed especially from large, long-term cohort studies involving COVID-19 patients, to establish or reject this pathogenetic relationship.

## 1. Introduction

As of 31 January 2023, the severe acute respiratory syndrome coronavirus 2 (SARS-CoV-2) infected more than 750 million people, causing more than 6 million deaths around the world [1]. The COronaVIrus Disease 19 (COVID-19), the respiratory disease induced by the virus, has a broad range of manifestations, elapsing without any or mild upper respiratory tract symptoms such as fever, dry cough, headache, and the loss of smell and taste, or provoking an interstitial pneumonia that can evolve into ARDS (Acute Respiratory Distress Syndrome), demanding urgent mechanical ventilation [2,3]. Among hospitalized patients, cough (70–80%), shortness of breath (50–60%), myalgia or fatigue (40–50%), and fever (90–98%) are the most frequent presenting symptoms. Moreover, 20–30% of patients develop gastrointestinal manifestation with nausea, vomiting, and diarrhea. Infrequent sputum production, headache (8%), and hemoptysis have been reported. The lag time from the symptoms’ onset to dyspnea is 5.0 days, while to hospitalization is 7.0 days and to ARDS is 8.0 days [4,5]. Around 20–30% of patients required an intensive care unit (ICU) setting for ventilatory support: patients treated in the ICU are generally older (median age, 66 years vs. 51 years) and have a higher burden of comorbidities, such as cardiovascular diseases (10–15%), hypertension (15–25%), obesity, diabetes (20–25%), or chronic obstructive pulmonary disease (COPD). Blood tests usually show leukopenia (20–40%) with lymphopenia (20–45%) and increased levels of aspartate aminotransferase (40%). Bilateral patchy shadows or ground glass opacity in the lungs are detected in chest computed tomographic (CT) scans of all patients [6]. A Chinese study confirmed that the chest CT scan is more sensitive and faster than PCR, even if it is less specific, for the diagnosis of SARS-CoV-2 infection [7]. For the first year of the pandemic, therapies were principally symptomatic and supportive. Later, new antiviral drugs and, above all, primary prophylaxis with vaccines [8], including first-time approved mRNA-based technology vaccines (Pfizer-Biontech’s BNT162b2 and Moderna’s mRNA-1273) [9,10], have changed radically over the course of COVID-19. They, in fact, elicit strong humoral responses and have shown to be safe in the majority of immunized people [11,12]. As of the end of January 2023, in the European Union, more than 970 million doses have been administered [13]. 

The endocrine system and, particularly, the thyroid gland, can be affected from the SARS-CoV-2 virus either directly, as with other post-viral subacute thyroiditis, or indirectly, via the systemic immune activation and “cytokine storm”, whose pro-inflammatory cytokines can induce detrimental consequences on thyroid function. Additionally, COVID-19 seems to be followed by both new onset and the recurrence of Graves’ disease (GD) and Hashimoto’s thyroiditis (HT), raising concerns about the virus’ potential role in triggering autoimmunity. Thus, with this review, we aimed to summarize the current knowledge regarding the potential causal relationship between SARS-CoV-2 infection and autoimmune thyroid diseases (AITD).

## 2. Method of Literature Search Statement

A literature review was conducted to identify all published studies (case series, original articles, and reviews) regarding the relationship between “thyroid” and “SARS-CoV-2” and/or “COVID-19” using the PubMEd/MEDLINE database, from the inception to July 2023. The titles and abstract were initially screened. Full-text articles were obtained for all potentially relevant articles and read for inclusion and data collection. References in the full-text articles were screened for relevance. 

## 3. The Virus Entry inside the Cells

The SARS-CoV-2 coronavirus, the etiological culprit of COVID-19, can enter the cells via the angiotensin-converting enzyme 2 (ACE2) receptor. The virus surface displays the homotrimeric spike glycoprotein, constituted by the S1 and S2 subunits, which binds to ACE2 [14]. During this initial interaction, the S1 subunit is disconnected with the ACE2 receptor, with the necessary help of transmembrane serine protease 2 (TMPRSS2). The resulting conformational change gives stability to the S2 subunit, allowing for membrane fusion [15]. The ACE2 receptor is fundamental for SARS-CoV-2 to infect cells and, differently from other coronaviruses, it does not require further co-receptors for cellular entry, (i.e., dipeptidyl peptidase 4 or aminopeptidase N) [16,17]. Among human beings, ACE2 mRNA can be found in several tissues, including in endocrine organs, such as the ovaries, testes, pancreas, and thyroid. TMPRSS2 mRNA is also expressed in the thyroid gland, pancreas, testes, and ovaries [18]. Thus, the endocrine glands are fully exposed and vulnerable to SARS-CoV-2 infection and to subsequent dysfunctions because of COVID-19. 

## 4. The Thyroid Dysfunction and SARS-CoV-2 Infection

**Figure 1:**
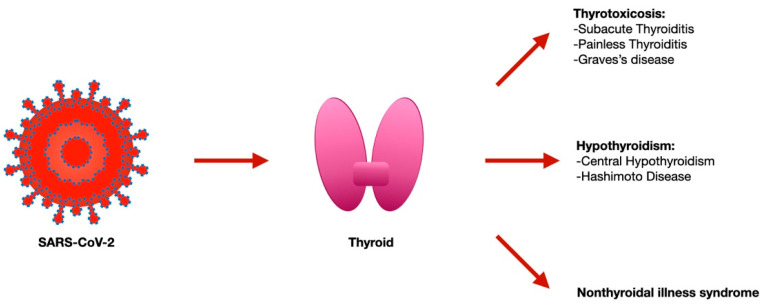
Thyroid disorders associated with SARS-CoV-2 infection.

The previous SARS pandemic already showed that affected patients had reduced thyroid function, and pathology investigations demonstrated that both thyroid follicular and parafollicular cells were extensively injured [19]. Since ACE2 mRNA, together with TMPRSS2, is expressed on thyroid follicular cells [20], the SARS-CoV-2 has the possibility to infect these cells as demonstrated via the autopsy report that found the viral genome and proteins inside these cells [21,22]. Several thyroid disorders have been reported with SARS-CoV-2 infection [subacute thyroiditis (SAT), thyrotoxicosis, and non-thyroidal illness syndrome (NTIS)] [23,24] and, in part, they are considered to be secondary to the local virus replication within the gland cells (Figure 1).

### 4.1. Acute Effects

Since the beginning of the pandemic, several cases of SAT were described [23,24,25]. In the ICU, patients with COVID-19 had a higher frequency of thyrotoxicosis and a lower TSH with respect to those admitted to a low-intensity ICU. However, during the 55 days of follow-up, none of them had ever complained about neck pain, but instead of lymphocytosis, they showed the typical lymphopenia of COVID-19 [26]. Among the COVID-19 patients not demanding intensive care management, overt thyrotoxicosis was detected in 10.8%, and hypothyroidism in 0.7%. Remarkably, thyrotoxicosis showed a correlation with serum interleukin (IL)-6 values, indicating that a greater inflammatory reaction exposed the patients to a higher chance of developing thyrotoxicosis [24]. On the other hand, most of the patients (74.6%) had TSH levels within the range [24], and this has been also confirmed via another sample of 334 patients with COVID-19, where 86.6% were euthyroid and none displayed overt thyrotoxicosis, even if those with COVID-19 had a lower admission of FT4 and TSH than those without COVID-19, according to an NTIS [27]. NTIS, previously known as euthyroid-sick syndrome, occurs during physiological stress, especially among hospitalized patients. It consists of an initial decrease in total T3 and fT3, paralleled by an increase in reverse T3 but not of TSH [28]. Long term and severe illness and ICU admission are associated with global reductions in TSH, fT4, and fT3 due to a fall in the hypothalamic thyrotropin-releasing hormone [29]. These thyroid function changes in the severe illness are considered protective from excessive tissue catabolism [30], and their magnitude varies with the severity of the illness: low serum T3 is associated with a longer hospital stay, ICU admission, and the need for mechanical ventilation in patients with acute heart failure [31], and it predicts a 30-day mortality in patients with community-acquired pneumonia [32]. For this reason, it is unsurprising that NTIS has also been detected among patients with COVID-19. The serum T4 levels also have an impact on the clinical outcomes of critically ill patients, and values lower than 3 mcg/dL have been associated with mortality rates in excess of 85 percent [33]. Nevertheless, critically ill patients with low serum T3 and/or low T4 do not appear to benefit from thyroid hormone replacement therapy [34]. COVID-19 pneumonia is associated with the reduced serum levels of TSH and total T3 with respect to other forms of pneumonia, but with no difference in total T4. These changes disappeared at recovery [27,35]. In one study, among 367 patients with a complete panel of TSH, fT4, and fT4, twenty-seven patients (7.4%) had NTIS, which was associated with a higher SARS-CoV-2 viral load and inflammatory markers [36]. Other studies have reported analogous data showing low TSH and/or low fT3 in patients with COVID-19 [37,38], with the amount of reduction related to the disease severity [38,39]. Furthermore, based on the results of the RECOVERY trial [40], glucocorticoids have become the standard of therapy for patients requiring oxygen supplementation and their use can lessen TSH and peripheral conversion of T4 to T3, contributing to the impairment of thyroid function, hence representing a potential confounder in the very beginning of the disease [41]. Notably, thyroid diseases and their specific therapies, whether for hypothyroidism or hyperthyroidism, when controlling for relevant confounding, seem to not have an impact on the risk and prognosis of SARS-CoV-2 infection [42,43].

### 4.2. Post-Acute Effects 

Although COVID-19 can impair the thyroid physiology during the acute phase of the disease, patients’ statuses returned to baseline following recovery. In 68 patients that healed from COVID-19, normal thyroid function was restored in 3 to 6 months after the acute illness, displaying TSH, fT4, or fT3 levels within range [44]. Moreover, Khoo and colleagues showed that 55 COVID-19 patients, whose TSH levels were also collected prior to the hospitalization (in 2019, before any cases of COVID-19), recovered to their baseline values after a median of 79 days of follow-up from the admission [27]. In addition, the long-term sequalae described in patients after acute SARS-CoV-2 infection, which is known as “post-COVID syndrome” or “Long COVID”, is characterized by a protracted course of various physical and neuropsychiatric symptoms, including fatigue, anxiety, low mood, sleep disturbance, breathlessness, myalgia, and “brain fog,” which have similarities to those induced by thyroid dysfunction. Therefore, such findings will have a significant impact since they would probably increase the demand of thyroid function assessment.

### 4.3. Autoimmune Thyroid Diseases 

More and more evidence suggests that SARS-CoV-2 is able to provoke the hyper-stimulation of the immune system, with the subsequent synthesis of several autoantibodies and the triggering of pre-existing or new onset autoimmune disease (AID), such as an antiphospholipid syndrome, autoimmune thrombocytopenia, autoimmune hemolytic anemia, and Guillain–Barre syndrome [45]. Moreover, we have observed that AID represents a predisposed condition to COVID-19; indeed, we found a significantly higher prevalence of COVID-19 in AID patients and an increased COVID-19-related mortality in systemic sclerosis (SSc) patients’ subgroup [46]. We have also found that AID patients that are not on conventional synthetic disease-modifying anti-rheumatic drugs (mainly hydroxyl-chloroquine and methotrexate) report a higher prevalence of COVID-19; this suggests some protective role of these drugs against the most worrisome complications of COVID-19 [47]. AITD are organ-specific autoimmune diseases mediated by T helper (Th)1 lymphocytes, whose main clinical manifestations are GD and HT, which can respectively lead to thyrotoxicosis and hypothyroidism. Numerous environmental risk factors (such as drugs, stress, radiation, seasonality, smoking, viruses, and iodine) are considered triggers of AITD in individuals with a genetic predisposition. The viruses may play a crucial role in the AITD onset via the activation of the adaptive and innate immunity [48]. Several viruses have been documented as a potential AITD culprit, such as the herpes simplex virus, Human T-cell lymphotropic virus-1, mumps virus, rubella, Epstein–Barr virus, enterovirus in HT, and retroviruses [human foamy virus or human immunodeficiency virus (HIV), human T-cell lymphotropic virus-1, and Simian virus 40] in GD [49]. Moreover, the human parvovirus B19 (EVB19) and Hepatitis C virus (HCV) have been associated with the development of AITD [48,49,50,51].

In fact, extrahepatic manifestations [52,53], including Sjogren’s syndrome, mixed cryoglobulinemia (MC), and endocrinological diseases (AITD and type 2 diabetes), are diagnosed in 38–76% patients affected by chronic hepatitis C (CHC) [54,55]. HCV seems to impair the self-tolerance (in immune cells or in thyrocytes) toward thyroid tissue, facilitating an autoimmune reaction against it in predisposed subjects [56,57,58,59,60,61,62,63]. HCV thyroid infection increases the synthesis of CXCL10 in thyroid cells, with the subsequent attraction of more Th1 lymphocytes into the gland [64]. Thyroid autoimmunity has also been reported significantly among MC + HCV patients versus the controls (AT 35 versus 16%; subclinical hypothyroidism, 11 versus 2%) (62). Notably, CHC patients, with or without MC but with AITD, showed an increased prevalence of PTC, suggesting that AITD might contribute towards TC development [65,66].

There are conflicting results about thyroid dysfunctions or the new onset of thyroid autoantibodies among patients recovered from COVID-19. While several reports state that these phenomena are infrequent and should consider a routine reassessment thyroid function test (TFT) among patients with initially normal TFT as not necessary [27,44,67], other researchers reported different results: in fact, Anaya and colleagues found that hospitalized patients with COVID-19 exhibited more frequent serologic thyroid autoimmunity than the pre-pandemic healthy controls (36.7% vs 20% *p* 0.007), suggesting that SARS-CoV-2 may be a trigger for AITD [68]. In another study, in 104 patients whose anti-thyroid antibody levels were remeasured after 3 months from their admission for COVID-19, increase in anti-thyroid peroxidase (AbTPO; *p* < 0.001) and anti-thyroglobulin (AbTg; *p* < 0.001) antibodies was observed, but not for anti-thyroid stimulating hormone receptor antibodies (*p* = 0.486). Among 82 patients with negative anti-TPO findings at the baseline, a significant interval increase in anti-TPO titer (by >12 U) was observed in 16 subjects, of whom four became anti-TPO positive. In this sample subset, the high C-reactive protein during hospitalization (*p* = 0.033), worse baseline clinical condition (*p* = 0.018), and higher baseline anti-TPO titer (*p* = 0.005) were associated with a significant increase in the anti-TPO titer. Nonetheless, it has been reported that 70% of this cohort of patients during the hospital stay have been treated with interferon beta-1b therapy, which likely confounded the investigation of the autoimmunity appearance along SARS-CoV-2-related thyroid dysfunctions [69]. To date, several new cases of GD [70,71,72,73,74,75], including with Graves’s ophthalmopathy (GO) [70], and of HT [75,76,77], following SARS-CoV-2 infection, have been reported in the literature. The time span between the infection and the onset of thyroid disease was heterogeneous, ranging from a concomitant appearance to more than 6 weeks, and all of these patients were managed with specific therapies. Several mechanisms have been hypothesized to explain the induction of autoimmunity elicited by SARS-CoV-2 infection: the immune system’s hyper-stimulation, the molecular mimicry between the self-antigens of the host and the virus, neutrophils extracellular traps, and finally, the virus induced the transcriptional changes in the immune genes (Table 1). Conversely, AITD seem to be associated with the augmented risk of contracting the infection. In fact, to explore this hypothesis, we conducted, during the first phase of the pandemic (from April to September 2020), an observational study that involved 515 consecutive unselected patients affected by thyroid disorders, 25 of whom had, confirmed (11/25), or highly suspected (14/25) SARS-CoV-2 infection [78]. The researchers separated the patients in two groups: patients with AITD (HT, Graves’ disease, patients with thyroid diseases, and circulating AbTPO and/or AbTg), and patients with no AITD. At the end of the survey, a higher prevalence of both symptomatic and asymptomatic COVID-19 was detected among the group of patients with AITD [78].

## 5. Potential Pathogenetic Mechanisms of SARS-CoV-2 Thyroid Autoimmunity Induction

### 5.1. Hyper-Stimulation of the Immune System by the SARS-CoV-2

Since the early phase of the pandemic, researchers have described the hyper-stimulation of the immune system promoted by SARS-CoV-2 [45]. In fact, the analysis of sera collected from the patients affected by COVID-19, from a mild to severe form of the disease, disclosed several changes in the peripheral leukocyte subpopulations and a significant surge in the levels of pro-inflammatory cytokines, particularly IL-6, IL-1β, IL-10, IL-17, TNF, and GM- CSF [79]. Primarily, IL-6 elicits robust pro-inflammatory activity binding the membrane receptor (mIL6-R) on the immune cells and its soluble receptor (sIL-6R), which chemoattract several other immune cells in the site of infection, promoting a rapid burst of the inflammatory response, consistent with the so-called “cytokine storm” or ‘cytokine release syndrome’. The ARDS itself and the hemophagocytic lympho-histiocytosis (HLH) reported worldwide in severely ill COVID-19 patients would be the expression of the hyper-stimulated immune system [80], which leads to an aggressive inflammatory response, noxious to the host tissue. Remarkably, ARDS and the subsequent respiratory failure are the main determinants of death in 70% of severely ill COVID-19 patients [81]. The cytokine storm also seems to be involved in the pathogenesis of the NTIS described in critically ill patients, as sustained by the in vitro and in vivo studies [82].

### 5.2. Molecular Mimicry between SARS-CoV-2 and Humans 

Historically, molecular mimicry, the recognition of a self-epitope as an external antigen because its similarity with pathogen-derived peptides, is considered one of the potential mechanisms by which an infection may influence the development of AID, including AITD. Recent results demonstrated a homology of several peptide sequences between human beings and SARS-CoV-2 proteins [83,84], and this similarity has not been shown in mammals not affected by SARS-CoV-2 [83]. Therefore, the adaptive immune system can synthetize antibodies against the virus components that may cross-react with self-antigens, thus leading to tissue pathology and potentially to AID’s new onset or relapse. Several human proteins, including thyroid-derived peptides, have been identified in share sequences with SARS-CoV-2 and likely becoming susceptible to the cross-activation of the autoreactive T and B cell by COVID-19, leading to severe autoimmunity. Studies reported that the principal proteins of SARS-CoV-2, including the spike protein, the nucleoprotein, and the membrane protein, all cross-react with TPO, due to the similarity and homology of their peptide sequences [84]. 

### 5.3. Neutrophils Extracellular Traps and SARS-CoV-2 Infection: Another Link with Autoimmune Responses

Neutrophils rule the innate immunity, also via the activation and release of extracellular traps (neutrophil extracellular traps, NET). NETs are characterized by extracellular fibers, which are principally composed of chromatin and DNA that are released from neutrophils and can bind the pathogens to be killed, without damaging the host tissues. However, NETs can also be involved in autoimmune conditions since they might contribute to self-antigens’ release. Indeed, excessive NET production is also related to the auto-inflammatory response, such as in systemic lupus erythematosus (SLE), rheumatoid arthritis (RA), myositis and multiple sclerosis (MS) [85,86,87]. SARS-CoV-2 pathogenesis has also been associated with an augmented NET formation and neutrophil-related cytokine responses [88]. Moreover, several clinical reports showed that patients infected with SARS-CoV-2 non-survivors show a progressive increase in NETs than the survivors [89,90]. Once activated, neutrophils release the NETs, which include not only histones, chromatin, and DNA, but also proteases and toxic enzymes, that increase the damage of the lung tissue and can directly lead to the deadly consequences of COVID-19. Some of the adverse outcomes registered with SARS-CoV-2-infection are coagulation dysfunction and widespread thromboses [91,92,93,94,95], which are events that are similar to those already observed in lupus patients [96].

### 5.4. Transcriptional Changes of Immune Genes

SAT is probably secondary to the virus replication within the thyroid gland [49], whereas NTIS and thyrotoxicosis are not strictly related to a direct virus entry [82]. Some studies have also shown that COVID-19 may come before the onset of GD, supporting the theory of the potential thyroid tropism of SARS-CoV-2 [70,71,72,73,74,75,97]. A recent study [98] reported an association between low-level enterovirus infection and GD via the decreased transcription of the type I interferon (IFN), the cytokine pathways, and the simultaneous increased activity of the NFKB1/RELA, JAK1/STAT1, IFNAR1, CCL2 chemokine, and IL18 genes, which have a crucial pathogenic role in autoimmune conditions [99,100]. The SARS-CoV-2 genome and its proteins have been recently found in the cells of testis, subcutaneous adipose tissue, and also, in the thyroid gland of patients who died from COVID-19, wherein viral proteins are expressed in the cytoplasm of thyrocytes [21,22]. It is worth mentioning the difference in the immune gene’s transcription between the virus-free thyroid of subjects with COVID-19 and the thyroid cells with evidence of SARS-CoV-2, which display a strong activation of the innate immune response, probably due to the up-regulation of both the type I (i.e., IFN-alpha) and type II (i.e., IFN-gamma) pathways. IFN-gamma is produced by innate lymphoid cells (ILC) and NK cells [101,102]; it has an important role in connecting the innate response with the adaptive one and in promoting the release of nitric oxide via macrophages [101]. The extended inflammation and damage involving the thyroid tissue during SAT may be attributable to the increase in the macrophage activity [49]. Many studies showed the essential role of the IFN-alpha and IFN-gamma pathways in contrasting the viral infection [101,103,104]; however, the extended and intense cytokines’ stimulation may result in damaging effects [101] that could lead to de novo recurrent autoimmune thyroiditis [101,105]. These findings suggest that COVID-19 may trigger or exacerbate thyroid autoimmunity in predisposed individuals, because once the virus replicates in endocrine cells, it may induce transcriptional changes in the immune genes, leading to the activation of the type I and type II IFN pathways [22]. 

## 6. Conclusions

To summarize, in a subset of patients, COVID-19 may acutely disrupt the thyroid function, either by subacute thyroiditis (with an atypical presentation, without the expected lymphocytosis and neck pain), NTIS, or even by triggering autoimmune disease. However, most patients are euthyroid since the infection onset and exploratory prospective studies suggest that when altered, the thyroid function recovers to the baseline, without specific therapy. For this reason, the meaning of such thyroid perturbations could be related to the more severe and unfavorable forms of acute COVID-19 [106]. However, infections could also act as environmental triggers of AID and AITD, and in months or years following SAT, a higher incidence of thyroid autoimmunity and hypothyroidism is reported [49,107,108]. After only three years, the pandemic ended, and the emerging data suggest that this phenomenon is also valid in the setting of SARS-CoV-2 infection, with the hypothesized pathogenetic mechanisms, here reviewed, including (a) the hyper-stimulation of the immune system, (b) molecular mimicry, (c) NETs, and (d) transcriptional changes in the immune genes. The development of these autoimmune sequalae will take time and large, long-term, follow-up studies of COVID-19 patients are needed to better characterize these disorders that could potentially affect several numbers of people.

## Figures and Tables

**Table 1:** Potential pathogenetic mechanisms of SARS-CoV-2 thyroid autoimmunity induction.

Immune System Hyperstimulation
Molecular mimicry Neutrophil extracellular traps (NETs) Transcriptional changes in the immune genes

## Data Availability

Not applicable.
